# Role of Autonomic Nervous System and Orexinergic System on Adipose Tissue

**DOI:** 10.3389/fphys.2017.00137

**Published:** 2017-03-10

**Authors:** Giovanni Messina, Anna Valenzano, Fiorenzo Moscatelli, Monica Salerno, Antonio Lonigro, Teresa Esposito, Vincenzo Monda, Gaetano Corso, Antonietta Messina, Andrea Viggiano, Antonio I. Triggiani, Sergio Chieffi, Giuseppe Guglielmi, Marcellino Monda, Giuseppe Cibelli

**Affiliations:** ^1^Department of Clinical and Experimental Medicine, University of FoggiaFoggia, Italy; ^2^Department of Experimental Medicine, Second University of NaplesNaples, Italy; ^3^Department of Medicine and Surgery, University of SalernoSalerno, Italy

**Keywords:** adipose tissue, autonomic nervous system, orexin, body composition, thermogenesis

## Abstract

Adipose tissue, defined as white adipose tissue (WAT) and brown adipose tissue (BAT), is a biological caloric reservoir; in response to over-nutrition it expands and, in response to energy deficit, it releases lipids. The WAT primarily stores energy as triglycerides, whereas BAT dissipates chemical energy as heat. In mammals, the BAT is a key site for heat production and an attractive target to promote weight loss. The autonomic nervous system (ANS) exerts a direct control at the cellular and molecular levels in adiposity. The sympathetic nervous system (SNS) provides a complex homeostatic control to specifically coordinate function and crosstalk of both fat pads, as indicated by the increase of the sympathetic outflow to BAT, in response to cold and high-fat diet, but also by the increase or decrease of the sympathetic outflow to selected WAT depots, in response to different lipolytic requirements of these two conditions. More recently, a role has been attributed to the parasympathetic nervous system (PNS) in modulating both adipose tissue insulin-mediated glucose uptake and fatty free acid (FFA) metabolism in an anabolic way and its endocrine function. The regulation of adipose tissue is unlikely to be limited to the autonomic control, since a number of signaling cytokines and neuropeptides play an important role, as well. In this review, we report some experimental evidences about the role played by both the ANS and orexins into different fat pads, related to food intake and energy expenditure, with a special emphasis on body weight status and fat mass (FM) content.

## Introduction

Adipose tissue, once seen as a mere passive reservoir for energy storage, today is considered a highly active endocrine and metabolic structure that react to over-nutrition with expansion and to energy deficit releasing lipids (Rutkowski et al., 2015). The primary cell type of adipose tissue are the adipocytes, which contains lipid droplets storing the excess calories, as triglycerides, without experience a lipotoxicity (Konige et al., 2014; Straub and Wolfrum, 2015). This unparalleled capacity to store and release lipids upon systemic metabolic demand links the cell biology of adipocytes and adipose tissue physiology to the whole-body metabolism. The adipose tissue can be divided into two subtypes, identified as white cells and brown cells. White adipose tissue (WAT) constitutes the typical fat cells: they are the majority of cells in both subcutaneous and visceral adipose depots. Brown adipocytes encompass smaller brown fat depots that play a role in thermogenesis in most mammalian species (Lapa et al., 2015; Rutkowski et al., 2015). The regulation of adipose tissue metabolism *in vivo* involves different central effector pathways, strongly influencing both energy intake and energy expenditure, and whose activity is regulated by adiposity-related signals.

Based on our experiences of studying human adipose tissue regulation *in vivo*, this review summarizes the complexity of said tissue function, with an emphasis on some peculiar aspects of the regulation of adiposity to disclose the integrative nature of adipose tissue function.

## Adipose tissue function and regulation

The different morpho-functional characterization of the adipocytes allows distinguishing adipose tissue as WAT and BAT. White adipose tissue has an evolutionary role permitting animals to survive for long periods without meals, thanks to their properties of energy storing, mainly as triglycerides, and of releasing fatty acids along fasting periods (Monda et al., 2008a; Messina et al., 2016a). Conversely, brown adipose tissue (BAT) is the most important organ for non-shivering thermogenesis (NST). The primary function of BAT is related to heat production in response to a decrease in core body temperature, due to environmental cold exposure. For this reason, brown adipocytes are characterized by an abundance of mitochondria, in contrast to white adipose cells. The thermogenic effect of BAT is strictly related to the presence of uncoupling protein 1 (UCP1), a transmembrane protein acting as mitochondrial metabolite transporter activated in the brown fat cell by fatty acids (Zingaretti et al., 2009; Yang and Ruan, 2015). Clusters of UCP1-expressing adipocytes with thermogenic capacity also develop in WAT in response to various stimuli (Vitali et al., 2012; Esposito et al., 2016). These adipocytes have been named “induced BAT.” The activities of brown and inducible “brown-like” adipocytes reduce metabolic disease, including obesity, in mice and correlate with leanness in humans.

Morphological differences between BAT and WAT are easily observable. In fact, BAT adipocytes have a polygonal shape, multilocular lipid droplets and a great number of large mitochondria. Moreover, they are innervated by numerous sympathetic nerve fibers (Zingaretti et al., 2009; Whittle et al., 2011; Villarroya and Vidal-Puig, 2013), confirming the central control of thermogenesis. Furthermore, to allow a high heat dissipation, BAT is also highly vascularized (Wu et al., 2013).

The modulation of both sympathetic and parasympathetic outflow provides a complex homeostatic mechanism enabling to specifically regulate the functional crosstalk of organs involved in the balance between caloric intake and energy expenditure (Villarroya and Vidal-Puig, 2013). Whereas the adaptive thermogenic response to cold and high-fat diet increases the SNS outflow to BAT, the different lipolytic requirements of these two conditions are appropriately met by the increase or decrease of the sympathetic outflow to selected WAT depots (Brito et al., 2008). A strict involvement of the SNS is also observed during caloric restriction, which is characterized by a decrease in the sympathetic outflow to BAT, resulting in a reduced energy expenditure and a simultaneous increase in SNS outflow to specific WAT depots facilitating lipid mobilization.

Signals related to food intake from various origins (e.g., gut, hepatic-portal area, baroreceptors) are integrated in the brain and result in increased peripheral sympathetic outflow. It is noteworthy to emphasize the role of diet composition in sympathetic responsiveness during the day, in view of the potential role of adrenergic per-responsiveness in the pathogenesis of obesity and metabolic syndrome (van Baak, 2008). It is well-known that chronic sympathetic hyper-responsiveness is present in central obesity; recent studies also demonstrate the consequence of a high sympathetic outflow to kidneys, heart, and blood vessels. Increased sympathetic reactivity can also be involved in the decline of insulin sensitivity, determining a vicious cycle responsible for hypertension, and the development of metabolic syndrome. Albeit the reason of this hyper-responsiveness is not yet clear, it may be driven by particular adipokines (Smith and Minson, 2012). While it has been clearly established that WAT receives sympathetic innervation, whether it receives parasympathetic innervation still appears to be controversial (Kreier et al., 2002). Neuroanatomical studies have demonstrated parasympathetic innervation of WAT in rats (Bartness, 2002). In addition, parasympathetic input affects hormone synthesis in WAT as evident from the effects of selective vagotomy on mRNA expression of resistin and leptin (Kreier et al., 2002; Di Bernardo et al., 2014).

**Figure 1:**
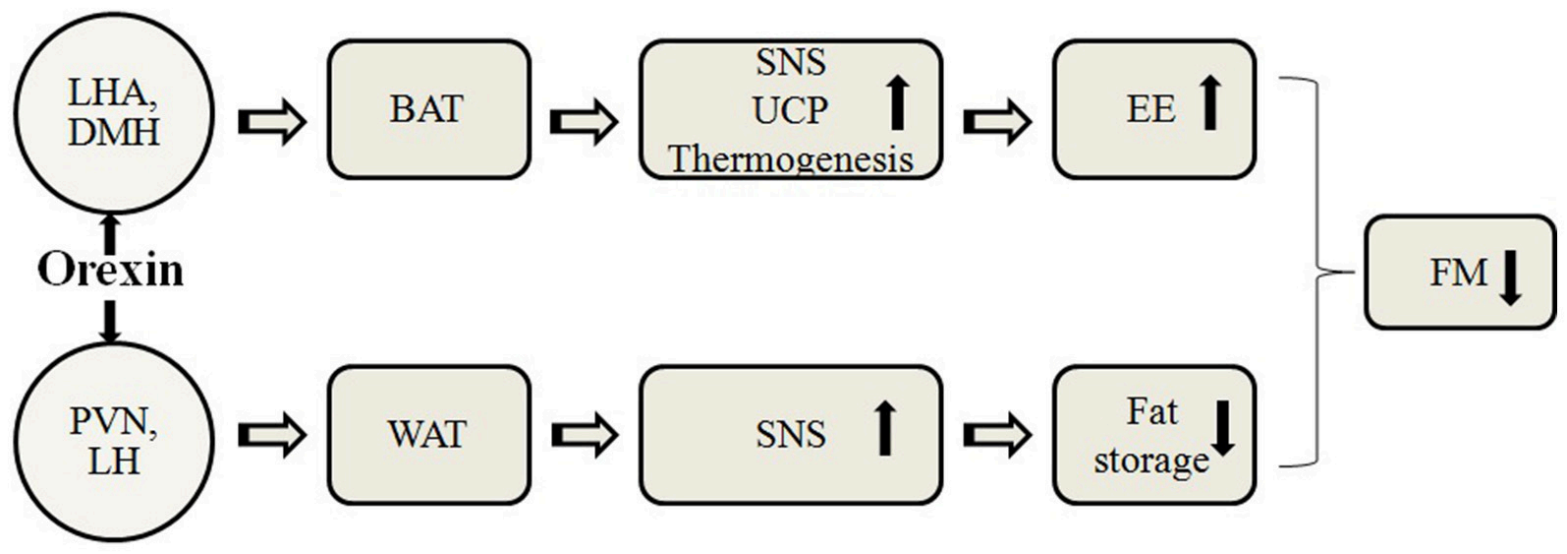
**A proposed model for action of central orexin on adipose tissue**. LHA, lateral hypothalamic area; DMH, dorsomedial nucleus of the hypothalamus; PVN, paraventricular nucleus of the hypothalamus; LH, lateral hypothalamus; BAT, brown adipose tissue; WAT, white adipose tissue; SNS, sympathetic nervous system; UCP, uncoupling protein; EE, energy expenditure; FM, fat mass.

Energy homeostasis is regulated by a complex network of neuroendocrine and autonomic pathways (Messina et al., 2014a), in which hypothalamus plays a key role monitoring signals that reflect energy status, thus initiating appropriate metabolic responses and behavioral (Suzuki et al., 2012; Esposito et al., 2014). The orexins (OX-A and OX-B), also named hypocretins (Eriksson et al., 2001; Messina et al., 2014b), are neuropeptides with critical functions in energy balance and obesity, and therefore in the accumulation of adipose tissue (Tsuda et al., 2002; Monda et al., 2008a). The neurons that produce those neuropeptides are in the lateral hypothalamic area (LHA), the dorsomedial nucleus of the hypothalamus (DMH), and the perifornical hypothalamus (Sakurai et al., 1998; López et al., 2010; Messina et al., 2016b). In this line, orexins play a crucial role in energy balance and feeding (Sakurai et al., 1998; López et al., 2010), and compelling evidence derived from genetic murine models suggest a role for orexins in promoting energy expenditure through modulation of locomotor activity and BAT thermogenesis (López et al., 2010; Sakurai et al., 1998). In fact, orexins are required for BAT development, differentiation, and function (Sellayah et al., 2011; Monda et al., 2014). Moreover, lack of orexins' action compromises energy balance, as demonstrated in orexin knockout mice, which are prone to diet-induced obesity, when compared with wild type mice (Shen et al., 2008; Sellayah et al., 2011). Figure 1 shows a proposed model for action of central orexin on adipose tissue (Monda et al., 2007). A protective role in aging-decreased thermogenic capacity was also recently suggested for orexins (Sellayah and Sikder, 2014). The aging process causes an increase in body fat percent, but the mechanism remains unclear. Aging is related to defective differentiation of BAT, alongside morphologic abnormalities and thermogenic dysfunction in humans and in rodents (Monda et al., 2006b; Sellayah and Sikder, 2014). In aged mice, indeed, interscapular BAT (IBAT) is progressively populated by adipocytes, bearing white morphologic characteristics (Monda et al., 2008b).

The orexin neurons have a wide projection pattern within the central nervous system, which includes several brain nuclei identified by WAT retrograde tracing studies (e.g., the arcuate nucleus of the hypothalamus, the locus coeruleus, the nucleus of the solitary tract and the suprachiasmatic nucleus (Peyron et al., 1998; Adler et al., 2012). Therefore, it is conceivable that orexin neurons may influence WAT metabolism, not only through their projections to the paraventricular nucleus of the hypothalamus (PVN), but also through modulation of other brain regions, suggesting that effects of orexins on WAT do not follow an exclusive pathway, but are distributed over multiple brain regions. Finally, as the orexin neurons appear to integrate multiple sources of metabolic and neuronal information (Kampe et al., 2009), it is possible that another role of these neurons is to coordinate the response from WAT based on the brain's perception of the metabolic status (Viggiano et al., 2016; Moscatelli et al., 2016a,b,c).

## Brown adipose tissue-mediated thermogenesis

**Figure 2:**
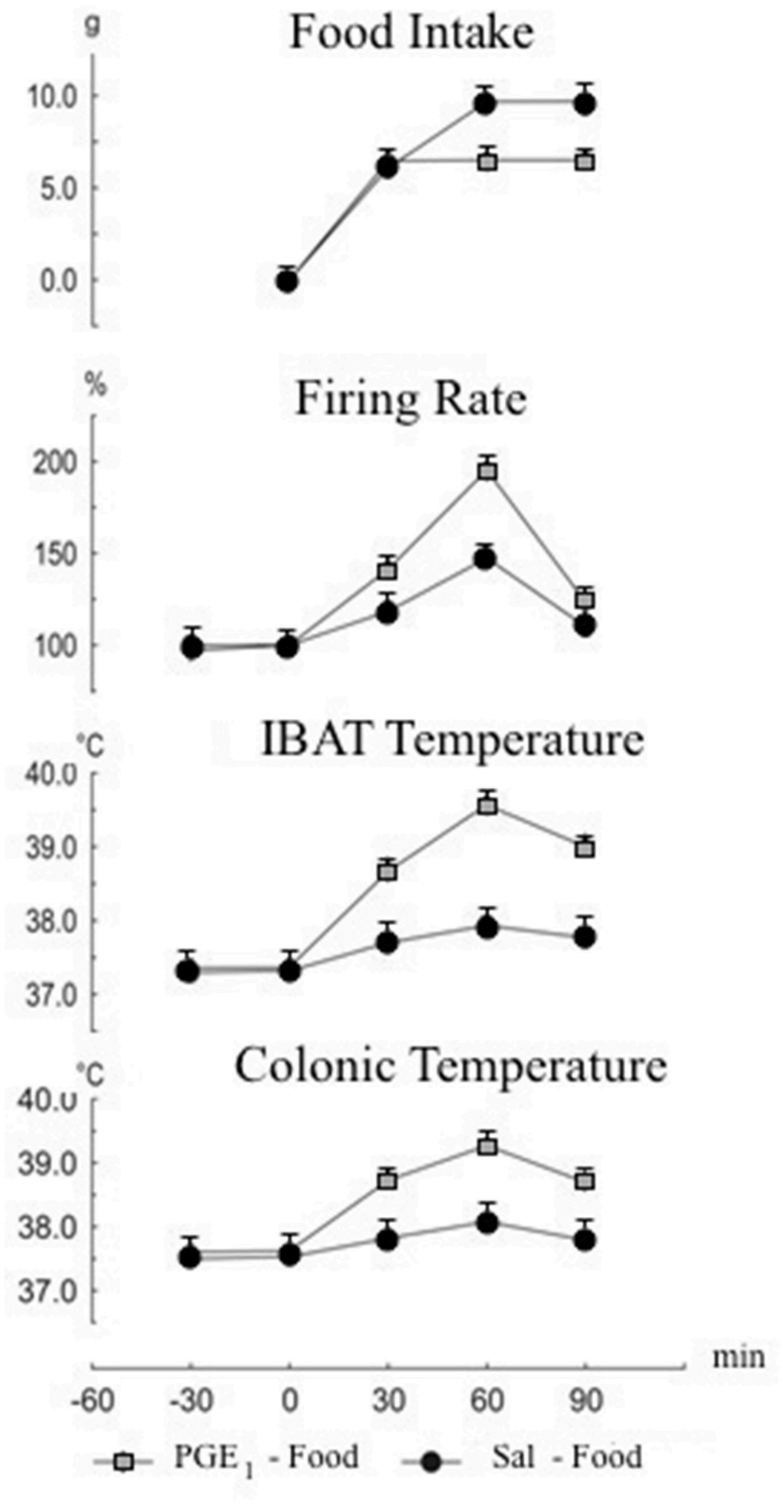
**Cumulative changes in food intake, firing rate of nerves to interscapular brown adipose tissue (IBAT), IBAT and colonic temperatures**. Food presentation at time 0. Intracerebroventricular injection of prostaglandin E-1 (PGE_1_) or saline was made at time 0. Values are expressed as mean ± standard error.

The IBAT is responsible for 35–65% of the total metabolic heat increase, unrelated to shivering in rodents (Rinaldi et al., 2015). *In vivo*, prostaglandin E_1_(PGE_1_) reduces heat loss and increases heat production in order to raise body temperature to a new set point. The simultaneous measurements of and food intake and sympathetic firing rate may represent the most relevant demonstration of the feed-back between body temperature and food intake, since the increase in body temperature due to PGE_1_ can be recognized as a signal of satiety, which reduces food intake (Mantzoros et al., 1996). Alterations of food intake and IBAT activity in response to hyperthermia induced by PGE_1_ injection in rat cerebral ventricle are reported (Monda et al., 1999). The firing rate of the sympathetic nerves to IBAT, along with IBAT and colonic temperatures were monitored in male Sprague-Dawley rats before and after food presentation. Saline or PGE_1_ were injected intraventricularly immediately before food presentation. The amount of food ingested was also measured. Prostaglandin E_1_ injection induced elevation of body temperature and reduction of food intake. Furthermore, IBAT temperature increase was inversely proportional to food intake. Figure 2 illustrates the cumulative effects of PGE_1_-induced hyperthermia. Overall, these findings provide direct evidence of sympathetic discharge of nerves to IBAT after PGE_1_ injection, supporting the hypothesis of a functional involvement of the ventromedial hypothalamus (VMH), a key structure in the control of sympathetic activity and food intake (Thornhill and Halvorson, 1994), as a consequence of the stimulatory effect of PGE_1_ upon the preoptic-anterior hypothalamus (PO/AH) (Thornhill and Halvorson, 1994).

## Interscapular brown adipose tissue activity and eating behavior

The PO/AH is considered the most important area deputed to control body temperature. The orexin A affects the temperature of IBAT, the most relevant effector of NST in rodents (Cannon et al., 1998), indicating that the rise in heat production is also due to a thermogenic mechanism independent of muscle contraction. Pyrogens, like PGE, influence PO/HA function inducing hyperthermia, whereas inhibitors of prostaglandins synthesis inhibit this response (Cannon et al., 1998).

**Figure 3:**
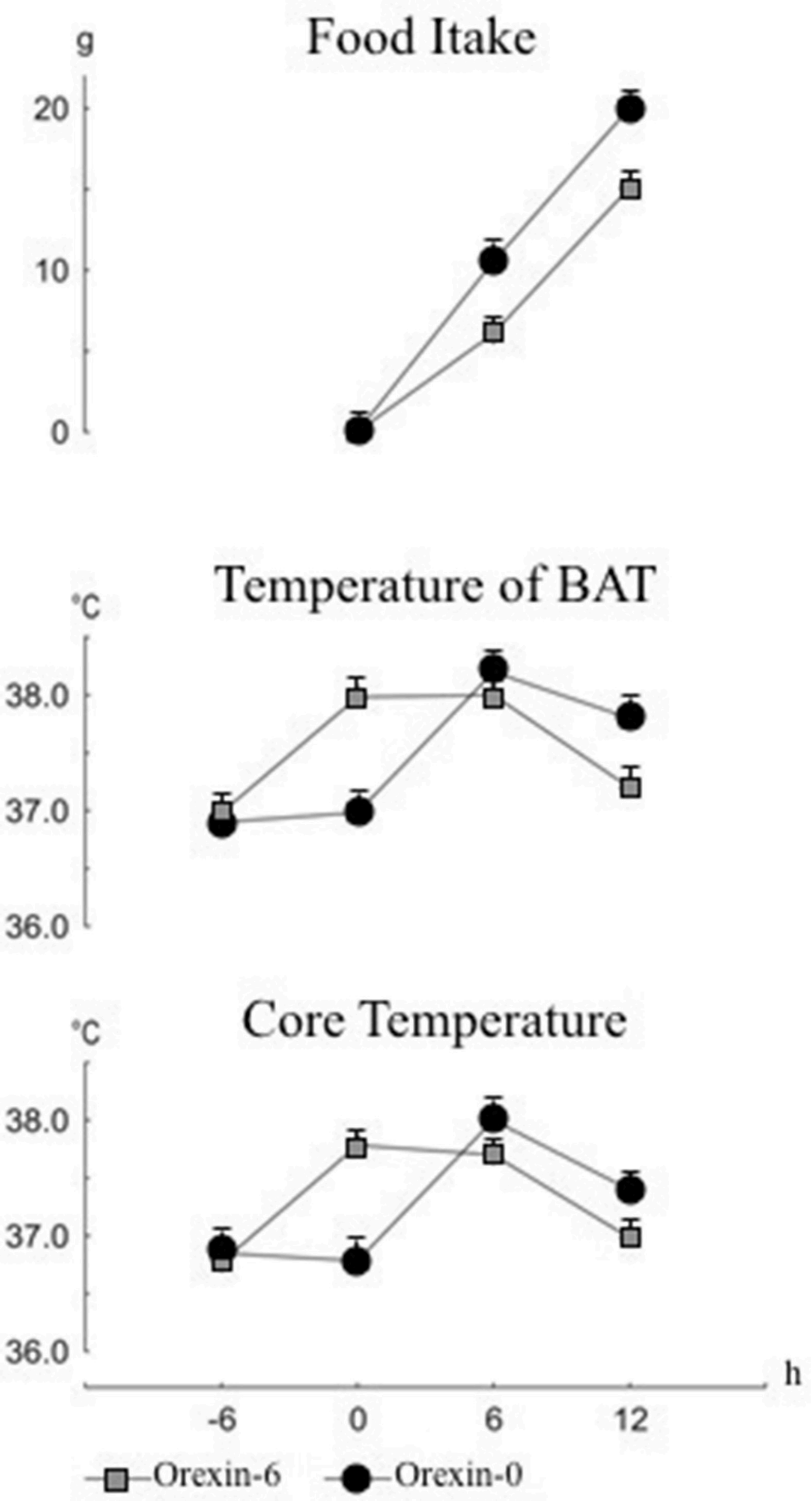
**Cumulative changes in food intake, temperature of interscapular brown adipose tissue (IBAT) and in core temperature**. Food presentation at time 0. Intracerebroventricular injection of orexin or saline was made at time -6 h or time 0. Values are expressed as mean ± standard error.

The effect of the thermogenic-induced orexin A activation upon eating behavior was investigated (Monda et al., 2013). Food intake, IBAT (T_IBAT_) and abdominal temperature (T_ab_) were monitored in 24-h fasting male Sprague-Dawley rats along 12 h after food presentation. Test animals received orexin A, through injection into the lateral cerebral ventricle, 6 h before food presentation. Control animals received orexin A contemporaneously to food presentation. As shown in Figure 3, a significant reduction of food intake, T_IBAT_ and T_ab_ was found in rats receiving orexin A prior to food presentation. These results clearly indicate that the reduction of food intake was a function of orexin A dependent temperature rise at the time of food presentation, outlining the prevailing role of orexin A in the control of body temperature, which in turn affects hypophagic behavior. According to these findings, the prevalent role so far displayed by the orexins in controlling eating behavior requires a substantial revision, since orexin A can induce simultaneously increase of sympathetic discharge, hyperthermia and hypophagia, thus contradicting the prevalent meaning of orexin as a primary hyperphagic substance. Conversely, other hyperphagic peptides, namely neuropeptide Y or galanin, induce a reduction of the sympathetic discharge and a decrease in body temperature (Bouali et al., 1995); while primary hypophagic substances, as leptin, cause an increase in the sympathetic activity and an increase in food intake (Haque et al., 1999).

## The relationship between heart rate variability and adiposity

Heart rate variability (HRV) power spectral analysis is widely considered a standard noninvasive method for assessing Autonomic Nervous System (ANS) function, due to its regulation of heart rate in a continuous, beat-to-beat manner (NASPE, 1996; Messina et al., 2012). Sympathetic activity is associated with the low frequency range (LF, 0.04–0.15 Hz) while parasympathetic activity is associated with the higher frequency range (HF, 0.15–0.4 Hz) of modulation frequencies of the heart rate (Messina et al., 2013). This difference in frequency ranges allows HRV analysis to separate sympathetic and parasympathetic contributions evident. Lower HRV is generally considered an indicator of poorer autonomic function (Viggiano et al., 2010; Messina et al., 2016c).

HRV was investigated in lean and obese women at premenopausal and postmenopausal age. As main findings, power spectral analysis of HRV showed a significant reduction in LF and HF components in obese than in lean subjects, both in premenopausal and postmenopausal age. These findings indicate a reduction of both the sympathetic and parasympathetic activity. The reduction of the sympathetic activity may play a key role in the weight maintenance in obese premenopausal women. Conversely, a reduction of the activity of sympathetic branch, could be linked to low energy expenditure, explaining the adipose tissue accumulation and the high body weight in premenopausal women. This is in line with the so-called “*Mona Lisa Hypothesis*,” acronym for “most obesities known are low in sympathetic activity” (Messina et al., 2013). Furthermore, the autonomic activity in postmenopausal women was lower than in premenopausal women, indicating that autonomic modulation changes in post menopause cannot be related to obesity. Several studies highlighted the relationship between an increase in sympathetic and thermogenic activity, and the reduction of food intake. So, it can be hypothesized that obesity can be related to the increase in food intake associated with a reduction of the sympathetic activity. On the other hand, some study pointed to a lower respiratory sinus arrhythmia, computed through the HRV power spectral analysis together with deep breathing tests, which indicated a cardiac vagal dysfunction in obese adolescents (Messina et al., 2012). Finally, a decreased parasympathetic activity may represent a final common pathway in different conditions related to higher rate of morbidity and mortality (Messina et al., 2012).

**Figure 4:**
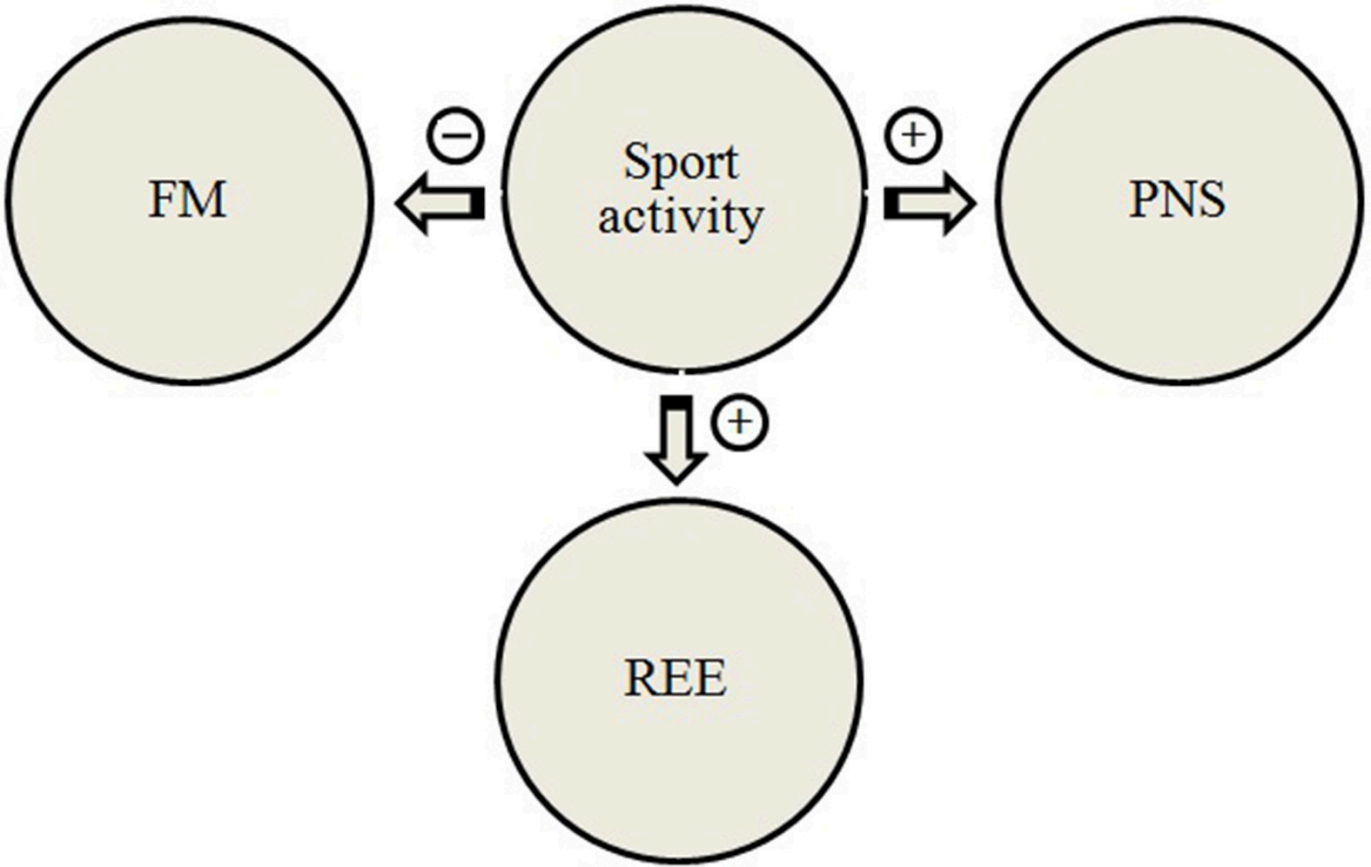
**A proposed model for the relationship between physical activity, resting energy expenditure, parasympathetic nervous system, and fat mass**. REE, resting energy expenditure; PNS, parasympathetic nervous system; FM, fat mass.

Physical training may induce several adaptive modifications, including changes in either ANS activity (Triggiani et al., 2015; Valenzano et al., 2016), or in resting energy expenditure (REE) (Kalsbeek et al., 2007). Heart rate variability power spectral analysis is an additional tool used to evaluate the autonomic hear rate control during exercise (Arai et al., 1989). The parasympathetic tone is enhanced by exercise training, so that a reduction in the heart rate, induced by vagal influence, is considered an index of training status in athletes (Kalsbeek et al., 2007). Moreover, body composition can be considered as a determinant for energy expenditure. The appraisal of the relationship among REE, tissue mass and HRV measures was carried out in adults female basketball players (Chieffi et al., 2004, 2012, 2014; Viggiano et al., 2014). Body composition, REE and HRV were measured before and after a period of 6 months in ten athletes and ten non-athletes. In athletes, physical activity induced an increase in REE and a decrease in FM, without any noticeable change in body weight. Athletes showed a significant increase in the parasympathetic activity, as revealed by the HF component of HRV. These findings showed a higher REE in athletes, than in non-athletes, despite the increased parasympathetic activity, typically related to lower energy expenditure (Viggiano et al., 2014). This is the first study examining the effects of long-term training on HRV, body composition, and REE. The relationship between physical activity, resting energy expenditure, parasympathetic nervous system (PNS) and fat mass is depicted in Figure 4. Furthermore, it is particularly relevant that exercise induced an increase of parasympathetic activity at rest, but the LF component of HRV did not show any change. Overall, the parallel increase in both parasympathetic activity and REE, found in long-term trained female athletes, can be considered an aspect of particular importance supporting the adaptive capacities of the athlete as compared to non-athlete. In fact, parasympathetic activity shows an inverse correlation with REE (Oldfield et al., 2007; Morrison et al., 2014).

**Figure 5:**
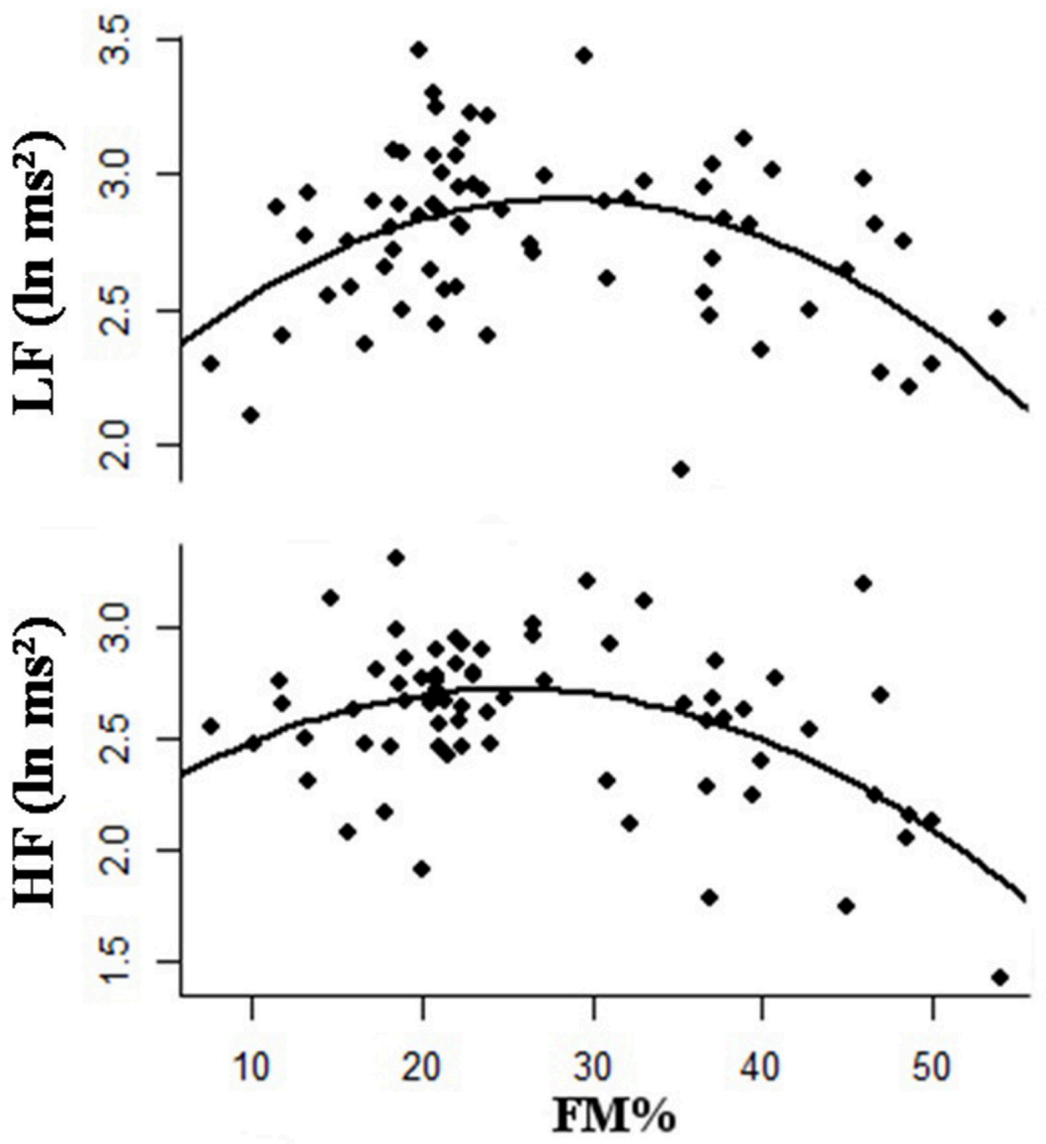
**Second order polynomial regression showing an inverted U-shaped relationship between percentage of body fat mass (FM) and low frequency power (LF) and high frequency power (HF)**.

Different patterns of adiposity are related to the occurrence of autonomic impairment and may be correlated with the observation of a higher risk for cardiovascular disease. The relationship between HRV and body mass index (BMI) has been repeatedly investigated in obese subjects with conflicting results (Zahorska-Markiewicz et al., 1993; Karason et al., 1999; Skrapari et al., 2007). The role of adiposity, measured as FM extent, on cardiac autonomic function was recently investigated in healthy adult women, by monitoring their short-term HRV response at rest (Triggiani et al., 2015). As a major finding, a reduction in both LF and HF bands, was found in overweight/obese, while in underweight subjects there was a reduction in the sole LF band. The simultaneous reduction of either HF, or LF components of HRV in overweight/obese women was related to a possible impairment in the baroceptive reflex sensibility. Conversely, in underweight women the reduction of the sole LF indicated that the baroceptive response was normal. More interestingly, the associations between body FM extent and HRV response was demonstrated adopting a curvilinear model, which indicated that a second order regression was considerably more successful to represent HRV changes, with respect to subjects' adiposity. The inverted U-shaped association (quadratic regression) between HRV and percent fat mass is shown in Figure 5. Overall, these data indicate that the adaptive flexibility of the autonomic cardiac activity found in both underweight and overweight/obese subjects, although differently reduced, turns out to be poorer, than in normal weight subjects. The question whether such a curvilinear model may reflect also differences in visceral fat distribution, is still opened.

## Discussion

The biological mechanisms underlying the precise regulation of eating behavior, energy expenditure, and energy storage in adipose tissue can be explained assuming that signals generated in proportion to body adiposity may influence the brain to modulate food intake and/or energy expenditure. Insulin has been the first signal molecule recognized to act in the brain to reduce food intake (Woods et al., 1979). Afterward, leptin was proposed as the principal responsible in this regulation process (Campfield et al., 1996). Although these two hormones probably play the major role in this regulation, an increasing number of endogenous signaling molecules, associated with neuroendocrine and autonomic control systems, are implicated as adiposity-related signals. The overall pattern of the central network innervating adipose tissue indicates sympathetic regulation of this tissue, in which orexins plays an important role. As reported in this review, the orexins are part of this network and can affect energy balance through modulation of either energy intake, or expenditure (Monda et al., 2008a). Fasting state is highly conditioning orexin receptors gene expression (Lu et al., 2000), a finding which has been confirmed by the functional interaction between orexin neurons and glucose-sensitive neurons in the hypothalamus (Shiraishi et al., 2000; Liu et al., 2001). More recently, Venner and coll. (Venner et al., 2011) proposed a role for orexin neurons as glucose sensors, since their electrical activity is dependent on intracellular energy levels changes in response to glucose concentration.

Sympathetic activation increases lipolysis and β-oxidation of fatty acids in BAT, allowing heat production, by drawing on lipids stores (Cannon and Nedergaard, 2004). Reduced thermogenesis, and thus lipid consumption, in BAT may contribute to the etiology of some forms of obesity. In fact, humans with low body temperature, maybe due to a low thermogenesis, are more inclined to obesity (van Marken Lichtenbelt and Daanen, 2003). In humans, obesity is associated with decreased BAT activity (van Marken Lichtenbelt et al., 2009).

An intra ventricular administration of orexin A induced an increase in firing rate of the sympathetic nerves to BAT, accompanied with a rise in BAT and colonic temperatures. The simultaneous increase in heart rate and body temperature after intra ventricular injection of orexin A shows a generalized activation of the SNS. Overall, the functional organization and neurochemical influences within the central nervous system networks govern the level of BAT sympathetic nerve activity to produce the thermoregulatory and metabolically driven alterations in BAT thermogenesis and energy expenditure, thus contributing to energy homeostasis (Morrison et al., 2014; Messina et al., 2015a).

Orexin A was shown able to influence both the thermogenesis and hyperphagia, so, the possibility that a previously activating thermogenic response orexin A might modify eating behavior was tested The result showed that the effects on orexin-induced food intake, depend on the time of food presentation. Such a result led us to review the functional meaning of orexin in food intake mechanism, highlighting the role of orexin A in the control of SNS activity and body temperature, which, sequentially, affects food intake (Monda et al., 2008b).

Food ingestion rise the body temperature caused by postprandial thermogenesis (Monda et al., 2008a; Messina et al., 2012). A reduced response of SNS might bring to an altered postprandial thermogenesis, becoming a crucial factor for obesity. Low postprandial sympathetic activation led subjects to a higher food intake to reach a prefixed level of body temperature. Conversely, overweight status increases the sympathetic discharge, and may contribute to induce diseases related to abnormal body weight (Lambert et al., 2010). Chronic sympathetic overactivity is well-known to play a role in central obesity, and many evidences demonstrate the consequence of a high sympathetic outflow to heart, kidneys, and blood vessels (Valenzano et al., 2016).

It has been generally assumed that obesity is characterized by the reduction of HRV reactivity, though these results are not even convergent. In previous studies, Monda et al. (2006a), demonstrated that the power spectral analysis of LF and HF components of HRV significantly changed in lean and obese women, due to the pre-/post-menopausal age. In obese pre-menopausal women, a lower sympathetic tone was found than in lean ones; while a parallel decrease in both LF and HF components appeared evident either in obese or in lean women after menopause. This unparallel effect of body fat content upon HRV variables in pre-/post-menopausal women indicates that the autonomic imbalance might rather be attributed to the age factor and the mutated sex hormonal balance, following the menopause onset. Indeed, suppression of sex hormones to postmenopausal levels reduces resting energy expenditure in young healthy women, through a reduction of autonomic nervous activity (Day et al., 2005; Messina et al., 2015b).

Heart rate variability is altered in obese subjects, but whether this is true also in underweight subjects is still under debate. In a recent study, we investigated the HRV profile in a sample of healthy adult women and its association with adiposity (Triggiani et al., 2015). The data reported in this study reflected the trend in HRV association with FM, among healthy adult women. In fact, a reduction in time and frequency domain measures was observed in overweight/obese women, when compared to normal weight subjects, which reflects sympathetic modulation of heart rate, in agreement with previously published studies. A similar HRV profile was found in underweight women, but the impairment of parasymphatetic activity was not proven. Our findings confirm that the adaptive flexibility of the autonomic cardiac activity found in both underweight and overweight/obese subjects, even though differently reduced, turned out to be poorer than in normal weight subjects. To look for associations between body FM extent and HRV indices we found that this relationship follows a parabolic trend, with lower HRV measures for either lower and higher FM values, leading to the question of whether this process might involve opposing or synergic processes that could be mediated by the two branches of the ANS. The different ways characterizing the power oscillatory RR signal reduction in underweight and overweight/obese subjects would suggest that a relationship between the autonomic bottom tone and the power of oscillatory RR signal, defined as *full scale effect*, may exists. Therefore, the underweight and overweight/obese status correspond to two distinct levels of tonic sympathetic activity, respectively, higher and lower than the level of tonic activity of normal weight subjects. In both cases, the expected response would be a reduced modulation of the sympathetic components in the LF band. Overall, these findings are consistent with previous studies demonstrating that the reduction of HRV in both underweight and overweight subjects may represent a risk factor for cardiovascular disease mortality (Nolan et al., 1998; Dudina et al., 2011).

## Conclusion

In this review, we have briefly focused on the relationship between ANS and orexins in the control of body weight, according to the theory of the “thermoregulatory hypothesis” of food intake. In summary, BAT is the strategic organ in the control of body temperature, through heat dissipation; while, WAT primarily stores energy as triglycerides and releases fatty acid during starvation. Although the central control of adipose tissue function was mainly based on the modulation of sympathetic outflow, recent developments have demonstrated that the orexins' system is a key factor in modulating adipose tissue functions by acting on several hypothalamic nuclei. Therefore, we hope that this review stimulates the reader's thinking to extend beyond the traditionally accepted roles of neural and hormonal factors in the control of body fat levels. To include the neural circuitry involved in orexin control of adipose tissue will help to provide therapeutic targets for obesity intervention.

## Author contributions

MS, TE, VM, and AM: conceived the study, participated in its design and wrote the manuscript. AVa, FM, AL, GCo, AM, AT, and SC: contributed to the conception and design. GM, AVi, GG, MM, and GCi: drafted the article and revised it critically for important intellectual content. GM: final approval of the version to be published. All authors read and approved the final manuscript.

### Conflict of interest statement

The authors declare that the research was conducted in the absence of any commercial or financial relationships that could be construed as a potential conflict of interest. The reviewer VP and handling Editor declared their shared affiliation, and the handling Editor states that the process nevertheless met the standards of a fair and objective review.
